# Machine learning reveals distinct temperature thresholds and environmental modulators for atopic dermatitis and allergic contact dermatitis prevalence in South Korea

**DOI:** 10.1371/journal.pone.0352199

**Published:** 2026-07-07

**Authors:** Ji Su Lee, Hyun Keun Ahn, Soo Ick Cho, Je-Ho Mun, Kyu Han Kim, Dong Hun Lee

**Affiliations:** 1 Department of Dermatology, Seoul National University College of Medicine, Seoul, Republic of Korea; 2 Department of Dermatology, Seoul Metropolitan Government – Seoul National University (SMG-SNU) Boramae Medical Center, Seoul, Republic of Korea; 3 Department of Dermatology, Seoul National University Hospital, Seoul, Korea; 4 InSkin Lab, Seoul, Republic of Korea; 5 Department of Dermatology, Veterans Health Service Medical Center, Seoul, Korea; Yonsei University Medical Center: Yonsei University Health System, KOREA, REPUBLIC OF

## Abstract

Atopic dermatitis (AD) and allergic contact dermatitis (ACD) are common inflammatory skin diseases influenced by environmental factors, but disease-specific environmental pathways remain poorly defined. This study developed a machine learning model to predict monthly disease prevalence and characterize distinct environmental conditions associated with each disease. We analyzed nationwide health insurance claims data for AD, ACD, and corns (control) from six major South Korean cities from 2012 to 2017, constituting 432 city-month records per disease. The M5P model tree algorithm predicted relative monthly prevalence based on meteorological data (temperature, humidity, precipitation, diurnal temperature range) and air pollutants (SO₂, NO₂, CO, PM10), with performance evaluated using Pearson Correlation Coefficient (CC) and Mean Absolute Error (MAE). Analysis of 3,990,692 AD and 16,890,182 ACD cases showed that the combined weather-pollution model achieved high accuracy for AD (CC = 0.839, MAE = 0.038) and ACD (CC = 0.932, MAE = 0.049). Mean temperature was the primary splitting variable for both diseases, but with different thresholds and secondary modulators. For AD, the initial split occurred at 17.4°C; above this, high PM10 (>44μg/m³) was associated with higher prevalence. For ACD, a notable split was identified at 11.65°C; below this, low humidity (<62%) appeared to be a key contributing factor. PM10 was a consistent predictor for both diseases. While temperature is a universal primary driver for both AD and ACD, the diseases follow distinct environmental pathways. AD is modulated by air pollution in warmer conditions, whereas ACD is sensitive to humidity in cooler conditions. This data-driven approach provides insights into disease-specific environmental triggers for public health interventions.

## Introduction

Atopic dermatitis (AD) is a common and chronic inflammatory skin disorder characterized by skin barrier dysfunction, immunological alterations, and pruritus [1]. The prevalence of AD has risen substantially over recent decades, now affecting up to 25% of children and 7–10% of adults worldwide. AD exhibits multifactorial pathogenesis characterized by immune dysregulation and skin barrier dysfunction as primary pathogenic mechanisms [2–5]. Clinical course variability is further influenced by diverse triggering factors including microbial infections, food and inhalant allergens, and broader environmental determinants. Allergic contact dermatitis (ACD) is another major inflammatory skin disease characterized by a delayed-type hypersensitivity response to exogenous agents. Like AD, ACD prevalence is rising and significantly influenced by environmental factors, with emerging evidence demonstrating that climate change and air pollution aggravate both conditions [6]. However, the interactions between these environmental factors are complex and controversial [7–11].

South Korea’s geographical position at the continental-oceanic interface creates a temperate climate with four distinct seasons and notable regional climatic variation, offering diverse environmental conditions for epidemiological analysis [12]. As a densely populated industrialized nation, South Korea faces significant air pollution challenges. Approximately 45% of the national population resides in six major metropolitan areas: Seoul, Busan, Incheon, Daegu, Daejeon, and Gwangju (S1 Fig and S1 Table). This high population density creates conditions where millions of citizens experience nearly identical weather and air pollution exposure. Notably, allergic skin diseases including AD and ACD show high and increasing prevalence in urbanized regions of East Asia, including Korea [13,14].

The National Health Insurance (NHI) system in Korea covered 97.2% of the population in 2013 [15]. Claims data included major diagnoses classified by the International Classification of Diseases, 10^th^ Revision (ICD 10) and the location of the medical institution visited. Korea’s mandatory NHI enrollment across all medical institutions ensures comprehensive disease surveillance at the regional level. Leveraging this extensive coverage with meteorological and air pollution data, previous studies have successfully identified environmental determinants of various health outcomes [12,16,17].

Machine learning, a subset of artificial intelligence, learns from training data to recognize patterns and generate predictions [18]. Machine learning algorithms, particularly decision trees, have been applied to analyze large-scale medical datasets including nationwide health records and air pollution data, yielding novel insights into cardiovascular disease, diabetes, and other conditions [19–21]. These methods identify complex non-linear associations and interaction effects within multidimensional environmental data, enabling population-level analysis that can uncover environmental determinants overlooked by conventional epidemiological approaches [22,23].

We employed the M5P model tree algorithm to overcome limitations of conventional statistical approaches. Unlike traditional regression requiring *a priori* specification of interaction terms, this data-driven method autonomously identifies clinically relevant environmental thresholds and models complex non-linear relationships through recursive data partitioning [24]. Using this approach, we developed predictive models for monthly disease prevalence to characterize the distinct environmental determinants of AD and ACD.

## Materials and methods

### Data source

The Monthly patient data for AD (ICD-10: L20.x) and ACD (L23.x) were obtained from the Healthcare Bigdata Hub provided by Korea’s Health Insurance Review & Assessment Service. Corn (L84) served as a control representing a non-eczematous skin condition. The analysis covered six major Korean cities (Seoul, Busan, Incheon, Daegu, Daejeon, and Gwangju) from January 2012 to December 2017. Weather data, including mean temperature, diurnal temperature range (DTR), relative humidity (RH), and precipitation, were retrieved from the Korea Meteorological Administration. Air pollution data for sulfur dioxide (SO_2_), nitrogen dioxide (NO_2_), carbon monoxide (CO), and particulate matter (PM10) were obtained from AirKorea. Detailed methodology is provided in the S1 Appendix. No missing values were identified in either the meteorological or air pollution datasets for the six cities over the study period. The disease records obtained from HIRA were also complete for all city-month combinations, with no inconsistencies identified.

The study was approved as exempt from review by the Institutional Review Board (IRB) of Seoul National University Hospital (IRB No. 1709-092-887). As this study involved secondary analysis of fully de-identified administrative claims data, the requirement for individual patient informed consent was waived by the IRB. The data were accessed for research purposes between March 2018 and September 2018. The dataset was fully de-identified prior to access, and the authors had no access to information that could identify individual participants at any time.

### Analysis of the impact of climate and weather on AD and ACD

The monthly patient numbers were adjusted according to the number of working days (weekday and Saturday) and holidays each month. First, a linear regression analysis was performed for each disease separately to assess the linearity between the number of patients per 1,000 and the number of working days or holidays in a particular month, while controlling for city, year, and month. (The monthly number of patients per 1,000 was calculated using the 2015 population data from the Korean Statistical Information Service, http://kosis.kr/). After adjusting for city, year, and month, the ratios of *β* coefficients of holiday/working day were 0.557 in AD, 0.698 in ACD, and 0.348 in corn, respectively. Based on these results, the difference in holiday/working day was compensated for each month. Finally, the relative monthly number of patients was calculated (S1 Appendix).

The model tree method was employed to construct a predictive model [24]. This approach combines decision tree learning with linear regression by recursively partitioning the data into subsets based on environmental variable thresholds, and fitting a separate multivariate linear regression model at each terminal node (leaf). Unlike many machine learning methods whose internal decisions cannot be directly inspected, this hybrid design is fully interpretable in that each decision threshold and its associated regression equation can be directly inspected by the reader, while autonomously identifying clinically meaningful environmental thresholds without requiring a priori assumptions. The M5P algorithm in the RWeka package (R statistical software version 3.4; http://www.r-project.org) was used with default pruning parameters. Specifically, a minimum of 4 instances per leaf node was required to prevent further splitting when a subset becomes too small. Standard post-pruning was then applied, whereby each subtree is replaced by a linear regression model at the corresponding inner node if the estimated prediction error of the subtree exceeds that of the node’s linear model, thereby removing branches that do not improve predictive performance and preventing overfitting. Finally, smoothed linear model predictions were generated by iteratively blending each leaf-node prediction with the linear models at successive parent nodes along the path to the root, weighted by the number of training instances at each node, to avoid sharp discontinuities between adjacent subtrees. The dependent variable was the relative monthly number of patients, and the independent variables were weather conditions (mean temperature, DTR, RH, and precipitation) and air pollution data (SO_2_, NO_2_, CO, and PM10). We compared the combined model (using both weather conditions and air pollution data) to individual models (using either weather conditions or air pollution data alone). Model performance was evaluated using Pearson correlation coefficient (CC) and Mean Absolute Error (MAE) (in units of relative monthly patient prevalence). K-fold cross-validation was conducted as follows: a part of the data (data from one year or one city) was designated as the test set and the remainder was set as the training set. A model trained on the training set was applied to the test set and evaluated by CC and MAE.

## Results

### Climatic and air pollution patterns in six cities

The analysis encompassed 432 city-month records (72 months across 6 cities from January 2012 to December 2017), with each record containing relative patient prevalence and corresponding environmental measurements for that specific city and month. Weather data were compared among the 6 cities (S2 Fig). The cities could be categorized as follows: high latitude (Seoul, Incheon, and Daejeon) or low latitude (Busan, Daegu, and Gwangju), and inland (Seoul, Daegu, Daejeon, and Gwangju) or coastal (Busan and Incheon). Cities at higher latitudes, such as Seoul and Incheon, showed a wider annual temperature range, while coastal cities like Busan exhibited a lower DTR. RH and precipitation were highest during summer. All air pollution values were lowest during summer (S3 Fig).

### Relative monthly number of AD and ACD patients

**Fig 1:**
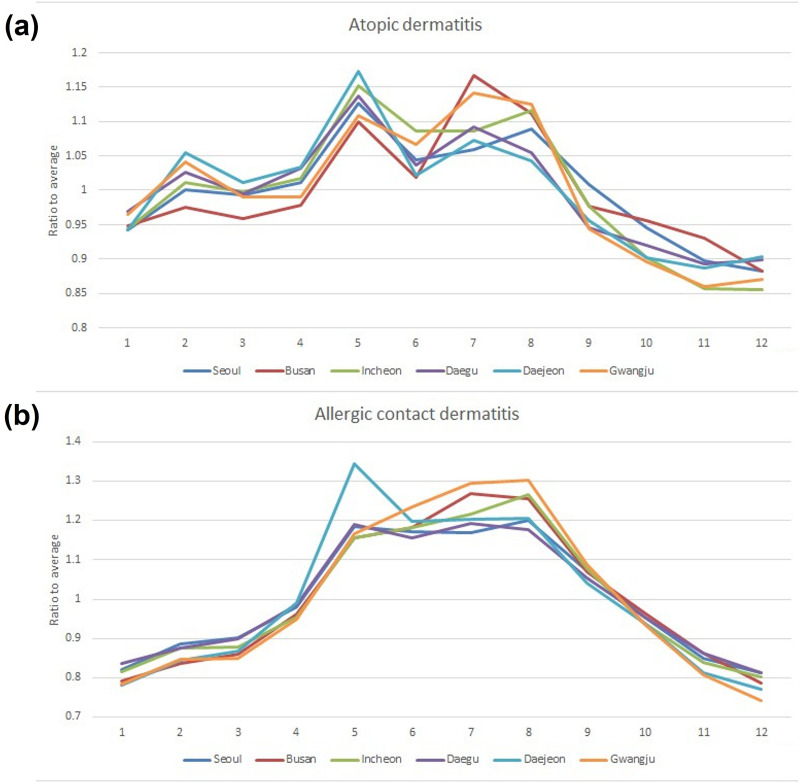
Relative monthly prevalence of atopic dermatitis and allergic contact dermatitis. Relative monthly prevalence of **(a)** atopic dermatitis and **(b)** allergic contact dermatitis patients across six cities – Seoul, Busan, Incheon, Daegu, Daejeon and Gwangju. The y-axis represents the ‘Relative monthly prevalence of patients’ as defined in the Methods.

The study cohort comprised 3,990,692 patients diagnosed with AD, 16,890,182 with ACD, and 1,162,722 with corn over the study period (from January 2012 to December 2017). The relative monthly prevalence of both AD and ACD patients showed seasonal patterns (Fig 1). AD prevalence showed two peaks, one in late spring (May) and a broader peak in summer (July-August). The seasonal fluctuation was considerably more pronounced for ACD than for AD. The relative monthly prevalence of ACD ranged widely from a winter low of approximately 0.75 to a summer peak of nearly 1.35, whereas AD prevalence showed more modest seasonal variation, ranging from approximately 0.85 to 1.15, suggesting a stronger link of ACD to seasonal environmental drivers.

### Impact of weather and air pollution conditions on the prevalence of AD and ACD

**Fig 2:**
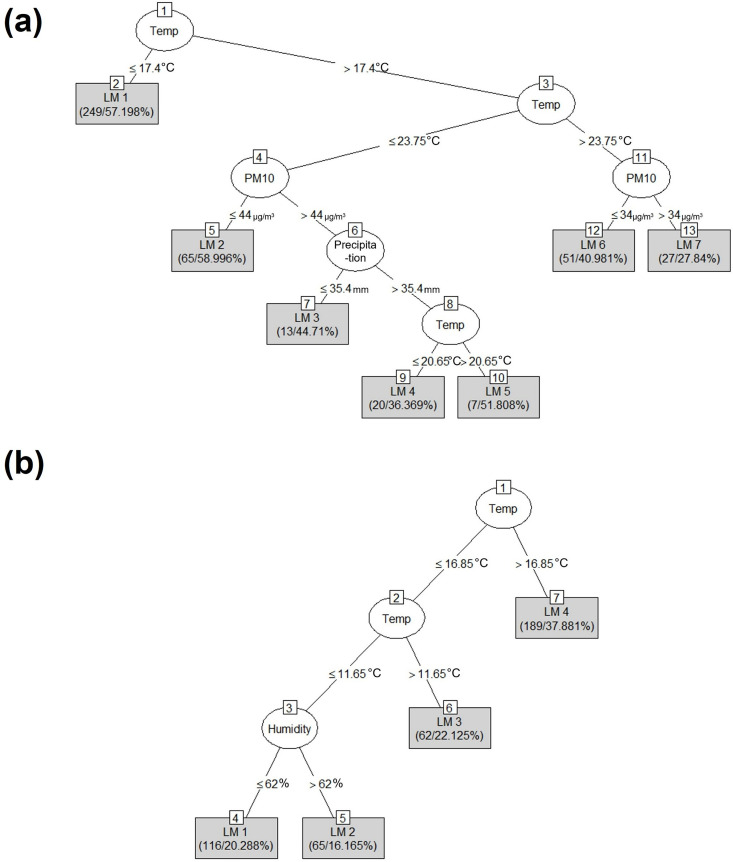
M5P model trees for atopic dermatitis and allergic contact dermatitis. The algorithm partitions the data at each node based on environmental variables and threshold shown. Each node shows the splitting variable and its threshold (e.g., Temp ≤17.4**°**C; PM10 > 44μg/m^3^; Precipitation >35.4 mm). Terminal leaves are linear regression models (LM). Each leaf node is labeled with the model number (e.g., LM 1), the number of city-month records it contains, and the percentage of the total dataset it represents (n/ %).

The M5P model tree method revealed different decision tree structures between AD and ACD (Fig 2). For AD, the model tree showed that mean temperature was the primary determinant of monthly prevalence, establishing an initial split at a threshold of 17.4**°**C (Fig 2a, Node 1). For cooler conditions at or below this threshold, the data, comprising the majority of observations (n = 249, 57.6% of the dataset), were classified into a single linear model, LM1 (Node 2). In this scenario, representing primarily the spring, autumn, and winter months, higher DTR was strongly associated with increased AD prevalence. Conversely, in warmer months with temperatures above 17.4**°**C, the model identified PM10 as the most critical secondary factor, with a threshold of 44 μg/m^3^ (Node 3). In these warmer months, when pollution was also high (PM10 > 44μg/m^3^), the data were further stratified by precipitation and temperature, leading to LMs 2–5 (Nodes 4, 6, 8). However, for the subset of data representing typical summer conditions (temperature >17.4**°**C and PM10 ≤ 44 μg/m^3^), the model was further split by a higher temperature threshold of 23.75**°**C to generate the final linear models (LMs 6, 7).

For ACD, mean temperature was also the primary splitting variable, but with different thresholds and secondary modulators (Fig 2b). The initial split occurred at 16.85**°**C (Node 1). In warmer conditions above this temperature, a large group of observations (n = 189, 43.8% of the dataset) was classified into a single linear model, LM4, where higher temperatures were strongly associated with increased ACD prevalence (Node 7). In cooler conditions at or below 16.85**°**C, the data were further stratified by a temperature of 11.65**°**C (Node 2). Within the coldest conditions (Temp≤11.65**°**C), relative humidity emerged as the key secondary factor, with a threshold of 62%. Under these conditions, low humidity (<62%) was associated with higher prevalence (LM1) compared to higher humidity conditions (LM2).

An examination of the coefficients within each linear model provides further detail on the influence of individual air pollutants (Tables 1 and 2). For both AD and ACD, PM10 was a consistently positive predictor across the majority of linear models, underscoring its role as a robust environmental contributor. For AD, SO_2_ also generally showed a positive association with prevalence. For ACD, CO was also a consistent positive predictor. In contrast, the coefficients for other pollutants, such as NO_2_ for both diseases, were inconsistent, showing negative values in some models (e.g., LM1, LM6, LM7 for AD). This may be attributable to multicollinearity among pollutants that share common emission sources, where the model statistically adjusts for their correlated effects. Therefore, PM10 stands out as the most reliable and consistently significant pollutant predictor in our analysis.

**Table 1:** Linear models (LMs) for atopic dermatitis generated by the M5P model tree algorithm.

	Constant	Mean temperature (°C)	Mean diurnal temperature range (°C)	Mean relative humidity (%)	Mean precipitation (mm)	SO_2_ (ppm)	NO_2_ (ppm)	CO (ppm)	PM10 (μg/m^3^)
LM1	0.7123	0.0004	0.0149	−0.0001	–	11.7028	−1.6712	−0.0827	0.0028
LM2	0.7889	0.0027	0.0013	−0.0001	−0.0002	0.6080	0.0240	0.0053	0.0046
LM3	0.9983	−0.0018	0.0047	−0.0001	−0.0001	0.6080	0.9168	0.0053	0.0019
LM4	1.0140	−0.0029	0.0038	−0.0001	−0.0001	0.6080	0.6643	0.0053	0.0019
LM5	0.9987	−0.0044	0.0038	−0.0001	−0.0001	0.6080	2.3134	0.0053	0.0019
LM6	0.6843	0.0129	−0.0014	−0.0001	−0.0000	7.9880	−3.7709	0.1683	0.0014
LM7	1.2717	0.0060	−0.0225	−0.0020	−0.0001	2.9690	−3.4536	0.0053	0.0016

The coefficients represent the change in the relative monthly number of patients for a one-unit increase in the corresponding variable, within the subset of data defined by that linear model. DTR, diurnal temperature range; RH, relative humidity.

**Table 2:** Linear models (LMs) for allergic contact dermatitis generated by the M5P model tree algorithm.

	Constant	Mean temperature (°C)	Mean diurnal temperature range (°C)	Mean relative humidity (%)	Mean precipitation (mm)	SO_2_ (ppm)	NO_2_ (ppm)	CO (ppm)	PM10 (μg/m^3^)
LM1	0.7634	0.0054	0.0040	−0.0002	−0.0002	−6.4075	0.0304	0.0096	0.0022
LM2	0.8265	0.0075	−0.0040	−0.0016	−0.0000	5.0629	−2.2755	0.0096	0.0028
LM3	0.5903	0.0111	0.0079	−0.0002	–	0.4242	1.4992	0.0096	0.0021
LM4	0.0802	0.0290	0.0138	0.0013	–	10.7055	−2.517	0.0121	0.0054

The coefficients represent the change in the relative monthly number of patients for a one-unit increase in the corresponding variable, within the subset of data defined by that linear model. DTR, diurnal temperature range; RH, relative humidity.

### Validation of linear models of AD and ACD

**Fig 3:**
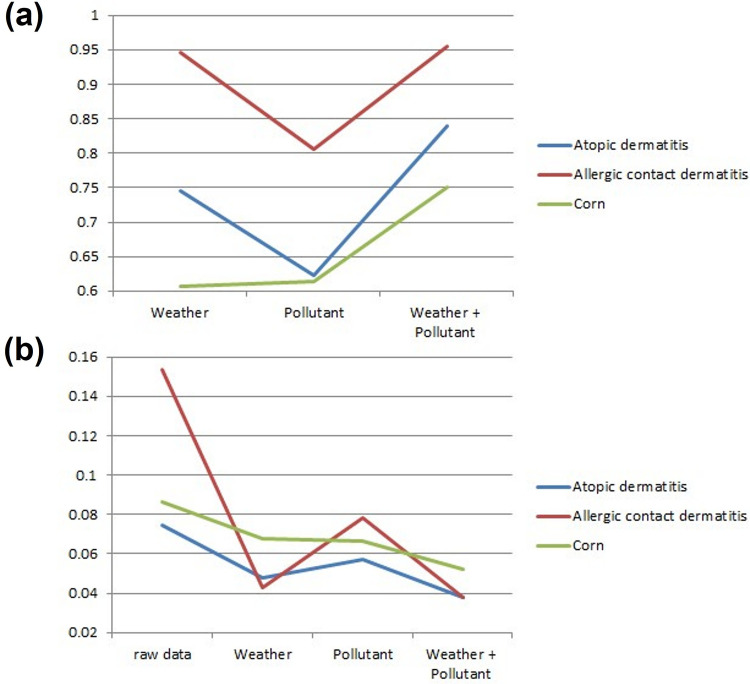
Performance of the combined linear models. Performance of the combined linear models for atopic dermatitis, allergic contact dermatitis and corn by the M5P model tree method, evaluated by **(a)** Pearson Correlation Coefficient (CC) and **(b)** Mean Absolute Error (MAE).

A combined model using both weather and air pollutant data performed better than a model using weather or air pollutants alone for all diseases (CC values were 0.839, 0.745, and 0.623, respectively, and MAE values were 0.038, 0.048, and 0.057, respectively) (Fig 3). The performance values for AD were intermediate, positioned between the most predictable ACD model and the least predictable corn model (non-environmental control).

Further validation was conducted using 6-fold cross-validation (S4 Fig). The 6-fold cross-validation supported the robustness of the combined model, which consistently outperformed the weather-only and pollutant-only models across most cities and years. However, the analysis also highlighted a key regional exception: the model’s predictive accuracy for AD was notably lower when tested on Busan data (CC ≈ 0.55) compared to other cities (CC > 0.7 for Seoul, Incheon, and Daegu). In contrast, the ACD model demonstrated consistently high performance across all cities (CC > 0.9), indicating that its environmental drivers are more geographically uniform. Performance was also reduced in models for predicting the monthly number of patients for the year 2015.

## Discussion

In this nationwide study utilizing a machine learning algorithm, we successfully developed and validated a predictive model for the monthly prevalence of AD and ACD based on environmental data. Our principal finding is that the environmental drivers associated with these two common eczematous conditions are fundamentally distinct. The model revealed that while temperature is a universal primary driver for both, AD prevalence is associated with air pollution (PM10) in warmer conditions, whereas ACD is more sensitive to humidity in cooler conditions.

Previous studies on AD and weather or air pollutants have shown complex and conflicting results. Silverberg et al. reported that high temperature decreased childhood eczema prevalence [9], and other investigators also supported the inverse relationship between AD and temperature [25–28]. Conversely, a positive relationship was also reported between AD and temperature [29–32]. Kramer *et al*. proposed that two types of eczema in children (summer and winter types) exist [33], and others reported different seasonal patterns among patients with AD [34]. Regarding humidity, the protective role of humidity against AD was reported in different regions [9,26,28]. In contrast, a humid environment has also been positively associated with the risk of AD [25,29,31,32]. Other weather factors like DTR or precipitation were less frequently reported. Kim et al. found that the worsening of AD symptoms was associated with high DTR [28]. Precipitation or rainfall has been shown to be positively correlated with AD [9,28,32].

Various types of air pollutants are known to be related to AD, including SO_2_ [7,32,35], NO_2_ [7,28,32,35–40], CO [35,36,38,41] and PM10/PM2.5 [7,28,32,35,37,38,42]. Air pollutants can exacerbate immune dysregulation and skin barrier dysfunction in AD [43,44]. Specific types of pollutants are known to influence AD differently. For example, traffic-related air pollutants (NO_2_ and CO) were associated with flexural eczema [36], and PM2.5 exposure, but not NO_2_ or CO, was associated with adult AD [42]. Heterogeneous outcomes from previous studies were probably attributed to the disparity in study designs (cohort, cross-sectional, case-control or panel study, among others), variables (climate or weather factors, types of air pollutants, indoor or outdoor), outcome parameters (prevalence or symptoms), definitions of AD (atopic dermatitis, flexural eczema, or childhood eczema, and so on), or subgroups (age or ethnic group).

The present study showed that the prevalence of AD was affected by both weather conditions and air pollutants. The effects of humidity [9,26,28,35] and PM10 [7,28,35,38] on AD were consistent with previous reports. Low humidity exacerbates skin barrier abnormality and inflammation *in vivo* [45]. The association between DTR and the prevalence of AD showed different patterns depending on the mean monthly temperature threshold and showed a strong positive correlation when the mean monthly temperature was below 17.4**°**C (LM1). A previous study has reported that DTR aggravated symptoms of AD only when DTR was > 14**°**C [28]. In Korea, DTR is relatively high during autumn through spring, but relatively low during summer. Taken together, high DTR during autumn through spring was associated with increased prevalence of AD when the mean monthly temperature was below 17.4**°**C. Our model identified 17.4°C as a critical threshold for AD prevalence, corresponding to Korea’s spring and autumn transition periods when AD flares commonly occur. The findings suggest a “two season” pathophysiology for AD triggers: below this threshold, high diurnal temperature range emerges as the primary predictor, likely reflecting meteorological stress on compromised skin barriers, while above this threshold, pollutant-induced inflammation and barrier disruption (PM10 > 44μg/m³) may represent the dominant contributing factor [43,44,46].

ACD exhibited distinct patterns compared to AD in response to weather conditions. For example, the effect of humidity on ACD prevalence was similar to that observed for AD except in LM4 (mean temperature > 16.85**°**C) under high relative humidity conditions. The prevalence of ACD was positively associated with mean temperature in all LMs, especially in LM4. This finding is in line with clinical observations that ACD is often more readily provoked under hot and humid conditions [47]. These findings may be explained by increased sweating and enhanced percutaneous absorption of allergens at higher temperatures [31], as well as greater outdoor activities and allergen exposure during warmer seasons.

The comparison between the AD and ACD model trees reveals fundamentally different environmental etiologies. While both are temperature-sensitive, AD exhibits complex weather-pollutant interactions, whereas ACD appears to respond more strongly to temperature and humidity. The ACD model demonstrates a progressive increase in prevalence with temperature, particularly above 16.85°C (LM4), which is clinically plausible given that elevated temperatures increase sweating and enhance contact allergen penetration. In the coldest conditions below 11.65°C, low humidity (<62%) emerges as a critical factor (LM1 vs LM2), suggesting that barrier dysfunction from dryness may sensitize skin to contact allergens even in winter. This clear distinction—AD’s sensitivity to air pollution versus ACD’s sensitivity to humidity—is a major finding of our study, providing large-scale epidemiological evidence for their distinct pathophysiological responses to environmental challenges.

An examination of the linear model coefficients shows that PM10 was consistently linked to higher prevalence of both AD and ACD in all LMs. Other air pollutants affected AD or ACD to some degree, although no significant consistent pattern was revealed. PM stimulates the aryl hydrocarbon receptor and pregnane X receptor in the skin, leading to exacerbation of inflammation [3,44,46]. Traffic-related air pollution like NO_2_ was linked to single nucleotide polymorphisms in AD [48], suggesting individual susceptibility [35]. The effect of air pollutants on AD and ACD suggests that PM_10_ was associated with the development of non-atopic eczema as well as AD.

The comparative model performance provides key insights into disease etiology. ACD demonstrated the highest predictability (combined CC = 0.932), indicating that monthly prevalence is predominantly driven by meteorological variables. Corns showed the lowest predictability (CC = 0.658), aligning with their non-environmental, mechanical etiology and serving as an effective negative control. AD’s intermediate predictability (CC = 0.839) quantitatively supports its multifactorial pathogenesis, where outdoor environmental factors contribute but share influence with non-modeled variables including genetic predisposition, indoor allergen exposure, and individual behavioral patterns [1,49].

The 6-fold cross-validation supported overall model robustness while revealing two notable exceptions. First, predictions for Busan consistently underperformed, suggesting this southern coastal city possesses distinct climatic characteristics not fully captured by our variables. Second, the model showed diminished performance for 2015, when the Middle East Respiratory Syndrome Coronavirus (MERS-CoV) outbreak disrupted healthcare utilization patterns in Korea [50]. The concurrent altered hospital-seeking behavior, quarantine measures, and anomalously low particulate matter concentrations likely confounded typical environment-disease relationships during this period.

Our study has several limitations. The diagnoses were based on claims data, and detailed clinical information such as the severity of AD and individual susceptibility could not be obtained. A specific limitation concerns potential misclassification of ACD (L23) in claims data, as xerotic or irritant contact dermatitis may be miscoded as L23 under cold, dry conditions. While the overall seasonal pattern of ACD (with prevalence peaking in summer rather than winter; Fig 1), the humidity-stratified decision tree structure, and the distinct behavior relative to the non-inflammatory control collectively argue against gross misclassification, future studies incorporating patch test-confirmed diagnoses would strengthen causal interpretation. Other potential confounding meteorological factors such as the ultraviolet index, or air pollutants such as PM2.5 were not included in the dataset due to unavailability. Additionally, seasonal biological allergens, including house dust mites and tree and weed pollens, were not included in the model. As these allergen cycles are temporally correlated with temperature and humidity, the observed meteorological associations may partly reflect unmeasured allergen exposure. Future studies incorporating biological allergen data would help disentangle direct environmental effects from allergen-mediated pathways. Regarding model generalizability, the model was validated internally using a K-fold cross-validation approach. External validation using independent datasets from other countries or climatic regions was beyond the scope of the present study, and future work should assess whether the identified environmental thresholds generalize to other geographic contexts. Lastly, although the reduced model performance in 2015 may have been influenced by the MERS-CoV outbreak, the potential effect of this structurally anomalous year on the identified environmental thresholds was not formally evaluated. Future studies should examine the stability of these thresholds after excluding years affected by major external disruptions.

However, this study has several strengths. Key strengths of this study include its hypothesis-generating capability, whereby machine learning autonomously identified clinically meaningful environmental thresholds and disease-specific pathways without *a priori* assumptions. Analysis of six metropolitan areas provided diverse climatic conditions and sufficient case volume for robust statistical analysis. Furthermore, the study revealed distinctive patterns differentiating AD and ACD from non-eczematous skin disease.

In conclusion, our findings indicate that AD and ACD are differentially affected by weather or air pollutant conditions based on specific environmental thresholds such as temperature, precipitation, and PM10. When counseling patients with AD and ACD, clinicians need to consider the effect of weather and air pollution on the prevalence and severity of these conditions. Future studies are warranted to establish more detailed models of AD and ACD, weather, and air pollutants based on data obtained under various conditions.

## Supporting information

S1 Fig Geographic locations of the six cities. Geographic locations of the six major South Korean cities included in the study, marked with yellow pins. Base map obtained from Google Earth (Google LLC); annotations added by the authors.
(TIF)

S2 Fig Meteorological data of six cities. Mean monthly (a) temperature, (b) diurnal temperature range, (c) relative humidity, and (d) precipitation of six cities – Seoul, Busan, Incheon, Daegu, Daejeon and Gwangju.
(TIF)

S3 Fig Air pollutants data of six cities. Mean monthly (a) SO_2_, (b) NO_2_, (c) CO, and (d) PM10 concentrations across six cities – Seoul, Busan, Incheon, Daegu, Daejeon and Gwangju.
(TIF)

S4 Fig Results of 6-fold cross-validation. Results of 6-fold cross-validation for atopic dermatitis, allergic contact dermatitis and corn showing (a) Pearson Correlation Coefficient (CC) and (b) Mean Absolute Error (MAE). The x-axis shows the test set used in each fold of the cross-validation (by city or by year).
(TIF)

S1 Table Population data of six cities. Each city has high population density and the four largest cities account for approximately 45% of the total population of the Republic of Korea. (Data quoted from Korean Statistical Information Service provided by Statistics Korea, http://kosis.kr/).
(DOCX)

S1 Appendix Supplementary Materials and Methods. (DOCX)
