# Supplementary figures and images for: Machine learning reveals distinct temperature thresholds and environmental modulators for atopic dermatitis and allergic contact dermatitis prevalence in South Korea
Source: PLoS One. 2026 Jul 7;21(7):e0352199. doi: 10.1371/journal.pone.0352199 (PMC13340855; doi:10.1371/journal.pone.0352199)

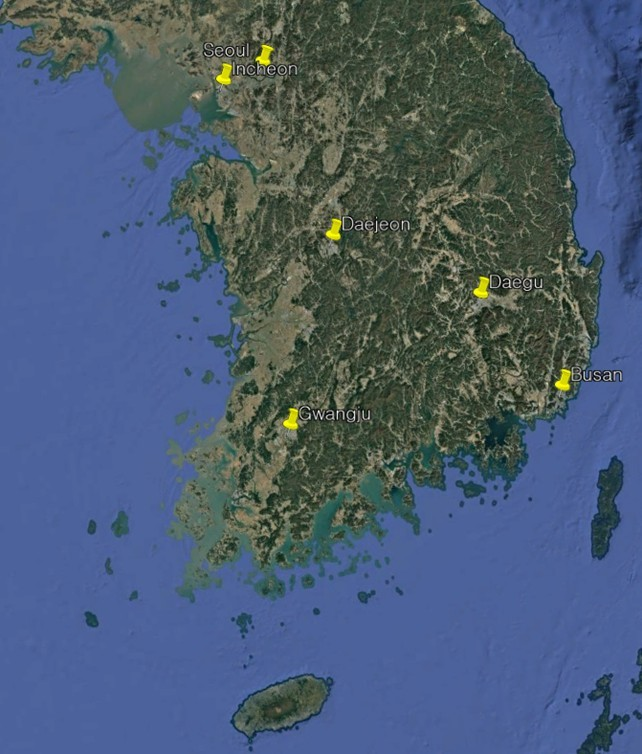

Supplement: S1 Fig — Geographic locations of the six major South Korean cities included in the study, marked with yellow pins. Base map obtained from Google Earth (Google LLC); annotations added by the authors. (TIF) [file pone.0352199.s001.tif]

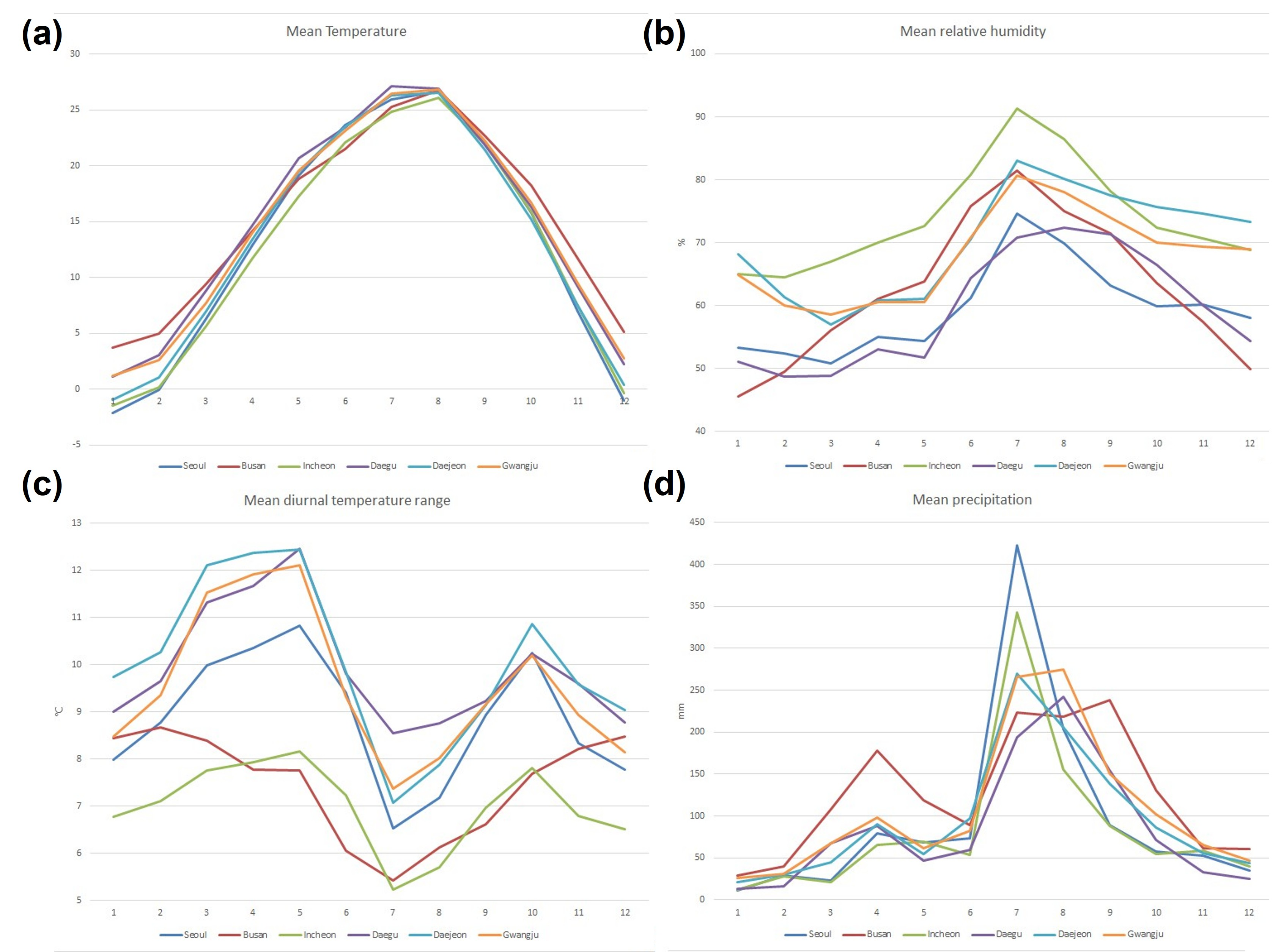

Supplement: S2 Fig — Mean monthly (a) temperature, (b) diurnal temperature range, (c) relative humidity, and (d) precipitation of six cities – Seoul, Busan, Incheon, Daegu, Daejeon and Gwangju. (TIF) [file pone.0352199.s002.tif]

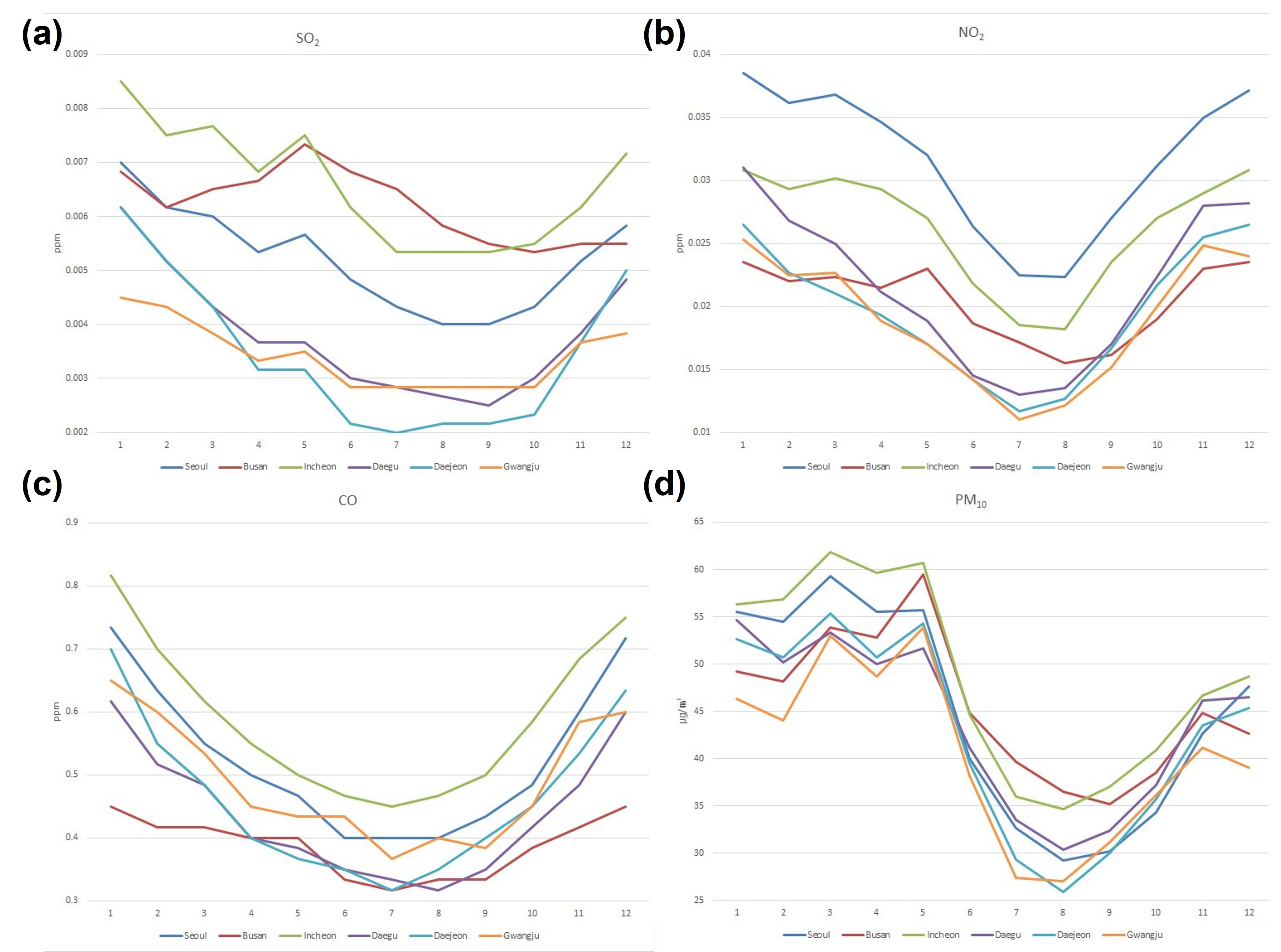

Supplement: S3 Fig — Mean monthly (a) SO2, (b) NO2, (c) CO, and (d) PM10 concentrations across six cities – Seoul, Busan, Incheon, Daegu, Daejeon and Gwangju. (TIF) [file pone.0352199.s003.tif]

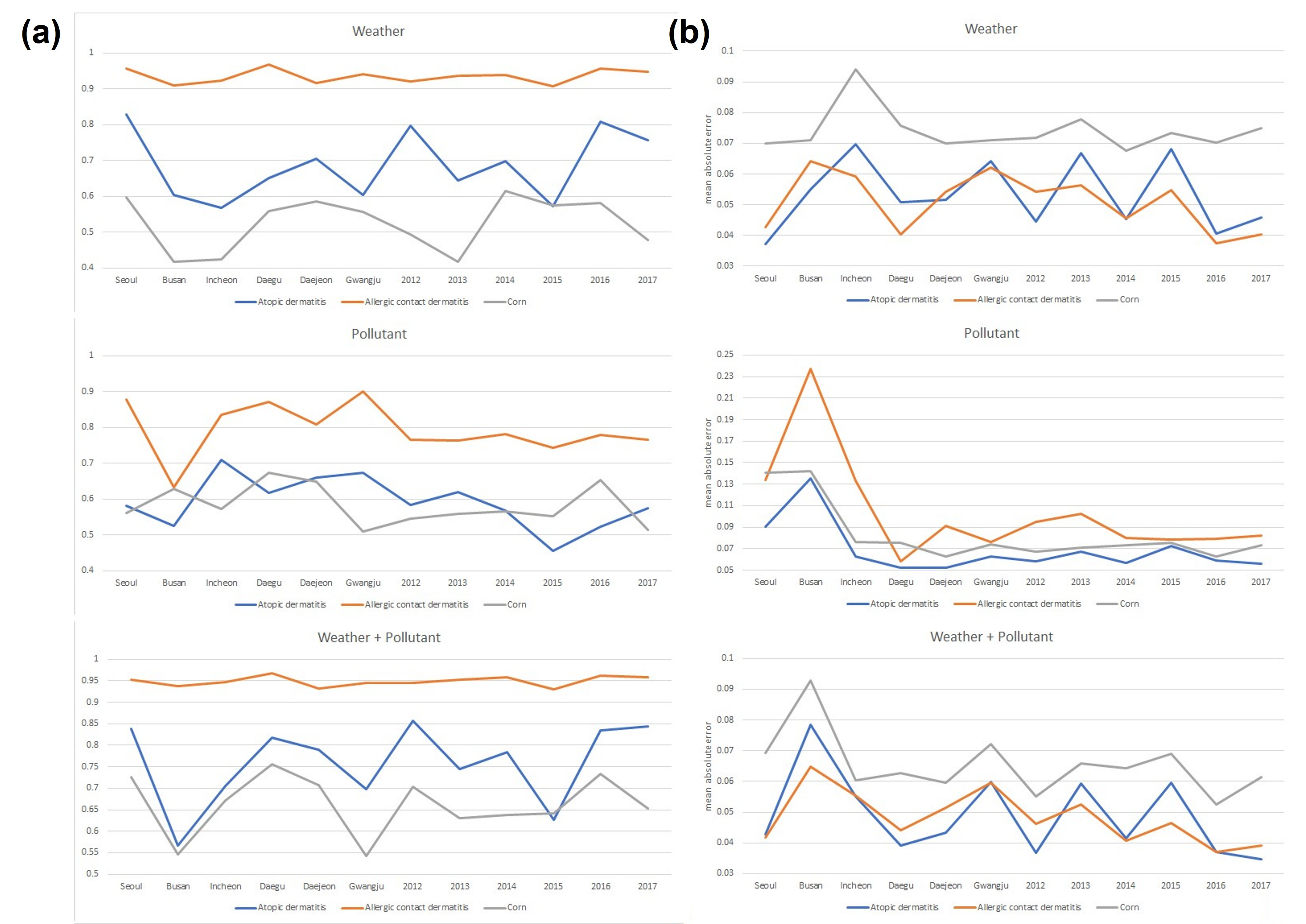

Supplement: S4 Fig — Results of 6-fold cross-validation for atopic dermatitis, allergic contact dermatitis and corn showing (a) Pearson Correlation Coefficient (CC) and (b) Mean Absolute Error (MAE). The x-axis shows the test set used in each fold of the cross-validation (by city or by year). (TIF) [file pone.0352199.s004.tif]
