# Supplementary material for: Machine learning reveals distinct temperature thresholds and environmental modulators for atopic dermatitis and allergic contact dermatitis prevalence in South Korea
Source: PLoS One. 2026 Jul 7;21(7):e0352199. doi: 10.1371/journal.pone.0352199 (PMC13340855; doi:10.1371/journal.pone.0352199)
Supplement: S1 Table — Each city has high population density and the four largest cities account for approximately 45% of the total population of the Republic of Korea. (Data quoted from Korean Statistical Information Service provided by Statistics Korea, http://kosis.kr/). (DOCX) [file pone.0352199.s005.docx]

**S1 Table. Population data of six cities.**

|  | **Seoul** | **Busan** | **Incheon** | **Daegu** | **Daejeon** | **Kwangju** |
| --- | --- | --- | --- | --- | --- | --- |
| Latitude | 37**° 34′** | 35**° 11′** | 37**° 27′** | 35**° 52′** | 36**° 21′** | 35**° 10′** |
| Population (2015) | 9,904,312 | 3,448,737 | 2,890,451 | 2,466,052 | 1,538,394 | 1,502,881 |
| Administrative area (2015) | 605 km^2^ | 770km^2^ | 1,049km^2^ | 884km^2^ | 539km^2^ | 501km^2^ |
| Population density  (2015) | 16,364.0 /km^2^ | 4,479.9 /km^2^ | 2,755.5 /km^2^ | 2,791.0 /km^2^ | 2,852.3 /km^2^ | 2,998.8 /km^2^ |

Each city has high population density and the four largest cities account for approximately 45% of the total population of the Republic of Korea. (Data quoted from Korean Statistical Information Service provided by Statistics Korea, <http://kosis.kr/>).
