# Supplementary material for: Machine learning reveals distinct temperature thresholds and environmental modulators for atopic dermatitis and allergic contact dermatitis prevalence in South Korea
Source: PLoS One. 2026 Jul 7;21(7):e0352199. doi: 10.1371/journal.pone.0352199 (PMC13340855; doi:10.1371/journal.pone.0352199)
Supplement: S1 Appendix — (DOCX) [file pone.0352199.s006.docx]

**S1 Appendix. Supplementary Materials and Methods**

***Data source***

The monthly numbers of patients with AD and ACD were collected from Healthcare Bigdata Hub (<http://opendata.hira.or.kr/home.do>), which is provided by the Health Insurance Review & Assessment (HIRA) Service in Korea. Monthly patient numbers in each specific city were classified according to ICD 10 codes for insurance claims. We chose L20.x (atopic dermatitis diagnosis code, including L20; atopic dermatitis, L20.0; Besnier’s prurigo, L20.8; other atopic dermatitis, and L20.9; atopic dermatitis, unspecified) and L23.x (allergic contact dermatitis diagnosis code) to compare AD and ACD. Corn (L84) was selected as a non-eczematous common skin disease unlikely to be influenced by weather or air pollution. The six largest cities in the Republic of Korea – Seoul, Busan, Incheon, Daegu, Daejeon, and Gwangju – were chosen as representatives. All data were extracted from January 2012 to December 2017.

Weather data were obtained from the Korea Meteorological Administration (<https://data.kma.go.kr/>), which included mean temperature (derived from maximum and minimum temperature), RH, and precipitation by month. The DTR was calculated by subtracting minimum from maximum temperatures. Air pollution data were retrieved from AirKorea (https://www.airkorea.or.kr), which provides monthly mean air pollution values including SO_2_, NO_2_, CO and PM10.

***Analysis of the impact of climate and weather on AD and ACD***

Computation of relative monthly number of patients:

$$\left( relative monthly number of patients \right) in a specific city in a specific year=\left( number of patients of a specific month \right) in a specific city in a specific year\div\left( mean monthly number of patients \right) in a specific city in a specific year)$$
